# *Stenotrophomonas maltophilia* Infections in Haematological Malignancies and Hematopoietic Stem Cell Transplantation: A Case Series including Cefiderocol-Based Regimens

**DOI:** 10.3390/medicina60010088

**Published:** 2024-01-03

**Authors:** Tommaso Lupia, Fabrizio Carnevale-Schianca, Davide Vita, Alessandro Busca, Daniela Caravelli, Elena Crisà, Vanesa Gregorc, Antonio Curtoni, Alessandro Cerutti, Nour Shbaklo, Silvia Corcione, Francesco Giuseppe De Rosa

**Affiliations:** 1 Unit of Infectious Diseases, Cardinal Massaia, 14100 Asti, Italy; francescogiuseppe.derosa@unito.it; 2 Unit of Oncology and Haematology, Candiolo Cancer Institute, FPO-IRCCS Strada Provinciale 142, 10060 Candiolo, Italy; fabrizio.carnevale@ircc.it (F.C.-S.); daniela.caravelli@ircc.it (D.C.); elena.crisa@ircc.it (E.C.); vanesa.gregorc@ircc.it (V.G.); 3 Department of Medical Sciences, Infectious Diseases, University of Turin, 10126 Turin, Italy; davide.vita@unito.it (D.V.); nour.shbaklo@unito.it (N.S.); silvia.corcione@unito.it (S.C.); 4 Department of Oncology, Stem Cell Transplant Center, Città della Salute e della Scienza Hospital, 10100 Turin, Italy; abusca@cittadellasalute.to.it; 5 Microbiology and Virology Unit, University Hospital Città della Salute e della Scienza di Torino, 10100 Turin, Italy; antonio.curtoni@gmail.com; 6 Intensive Care Unit, IRCCS Candiolo, 10100 Candiolo, Italy; alessandro.cerutti@ircc.it; 7 School of Medicine, Tufts University, Boston, MA 02111, USA

**Keywords:** *Stenotrophomonas maltophilia*, multi-drug resistance, haematological diseases, nosocomial pneumonia, bloodstream infections

## Abstract

*Background and Objectives*: *Stenotrophomonas maltophilia* is a ubiquitous, aerobic, Gram-negative bacillus causing increasing concern in patients affected by haematological malignancies. *Materials and Methods*: We report a case series from two centres in Northern Italy to describe the characteristics, outcome and microbiological response of *S. maltophilia* infections in patients with haematological malignancies and/or allogenic hematopoietic stem cell transplantation (aHSCT). *Results*: Ten patients were included. The median age was 67 years, and seven patients (70%) were males. The median Charlson Comorbidity Index was 6 (IQR: 4–8). The most frequent haematological comorbidities were acute myeloid leukaemia (AML; *n* = 3; 30%) and non-Hodgkin’s lymphoma (*n* = 3; 30%). Three (30%) patients underwent aHSCT before infection, all for AML. All the patients had undergone a recent antibiotics course and had an indwelling central venous catheter before infection. The main clinical presentations were nosocomial pneumonia, with (2; 20%) or without (4; 40%) secondary bloodstream infection and CRBSI (3; 30%). Four patients were treated with cefiderocol in monotherapy or combinations therapy with cotrimoxazole. The rest of the patients were treated with cotrimoxazole or levofloxacin in monotherapy. *Conclusions*: Despite a high rate of clinical improvement (90%) after starting antimicrobial therapy, we faced high 30-day mortality (30%) and in-hospital mortality (50%) rates in a highly comorbid population.

## 1. Introduction

*Stenotrophomonas maltophilia* is a ubiquitous, aerobic, Gram-negative bacillus first isolated in 1961 [1]; it has since been isolated in humans, animals and the environment [2]. Despite its low–moderate pathogenicity in immunocompetent hosts, it has inspired increasing concern in the past decades in patients affected by haematological malignancies [3]. This concern is heightened by the pathogen’s ability to form biofilms, producing site adhesion and facilitating the colonisation of hospital patients; hence, it is reported as a nosocomial pathogen [4].

*S. maltophilia* in haematological patients has two major clinical presentations of concern: catheter-related bloodstream infection (CRBSI) and haemorrhagic pneumonia (HP) [5], which is associated with a high mortality rate [6]. The risk factors for morbidity in patients colonised or infected by *S. maltophilia* include malignancy, respiratory diseases, prolonged hospitalisation, intensive care unit (ICU) admission, previous antibiotic treatment, and indwelling devices [4].

Because *S. maltophilia* is intrinsically resistant to most β-lactam drugs, including carbapenems and aminoglycosides, trimethoprim-sulfamethoxazole (TMP-SMX) is the drug of choice [7]. In a recent systematic review and meta-analysis, Maraolo and colleagues summarised the literature data regarding *S. maltophilia* treatment options [8]. As TMP-SMX has been associated with severe adverse effects caused by bone marrow suppression, the authors indicate that fluoroquinolones and tetracyclines may be reasonable alternatives to TMP-SMX [8], even though the former are frequently administered as antimicrobial prophylaxis during stem-cell transplantation (SCT) and chemotherapy, which can potentially lead to resistance or reduced effectiveness of fluoroquinolones against *S. maltophilia* [8]. Moreover, *S. maltophilia* can accumulate efflux pumps that confer resistance to fluoroquinolones and tetracyclines [9]. 

Among newly introduced therapeutic options, cefiderocol, a siderophore cephalosporin that is active against ESBL-producing Enterobacterales, carbapenems-resistant Enterobacterales and metallo beta-lactamases producers, also retains activity against *S. maltophilia*, including strains that are resistant to first line antibiotic options [10]. The FDA approved Cefiderocol in November 2019 for the treatment of cUTIs, and in September 2020, it was also approved for HAP and VAP [10,11]. In April 2020, the European Medicines Agency (EMA) approved cefiderocol for Gram-negative infections that have limited therapeutic options [11]. Alongside the interesting microbiological profile, cefiderocol is characterized by a good safety profile, potentially useful in high comorbid populations as well as haematological patients [12,13].

In this case, series we aimed to present data from two haematological centres in Northern Italy to describe clinical characteristics, outcome and microbiological response of *S. maltophilia* infections in patients with high-risk haematological malignancies and/or aHSCT. Moreover, we have collected data regarding treatments including cefiderocol-based regimens.

## 2. Materials and Methods

We reported a case series of *S. maltophilia* infections in patients hospitalised for haematological malignancies and/or HSCT. We describe our centres’ clinical, microbiological and treatment features of the infection cases. 

We enrolled patients hospitalized at IRCCS Candiolo Hospital and City of Health and Science University Hospital (Molinette Hospital) in Turin between 1 January 2021 and 1 June 2023. Medical records were revised independently by two authors (T.L. and D.V.).

The inclusion criteria were adults over 18 years old who had a previous diagnosis of haematological malignancy. Patients were included in the case series if they had been hospitalized during the period 2021–2023, and provided that they yielded *S. maltophilia* from at least one clinical specimen from their blood or respiratory tract, along with clinical signs of infection and the decision of the physician to treat for *S. maltophilia* upon identification of the pathogen. Inclusion criteria were stated by three authors (T.L., S.C. and F.G.D.R.)

Moreover, the exclusion criteria were patients without haematological malignancies, patients colonized by *S. maltophilia,* and those with no clinical features of infections. The distinction between colonization and infection with *S. maltophilia* is a critical issue; despite this, we have included patients whose clinical history and radiological, laboratory and microbiological features strongly suggest infection by *S. maltophilia*.

A total of 18 patients were enrolled in the study; subsequently, after the revision of medical records (during which records were revised independently by two authors, T.L. and D.V.), 6 patients were excluded for suspected colonization because of clinical history, radiological, laboratory and microbiological features not strongly suggesting *S. maltophilia* infection and according to the decision of the treating physician. Moreover, two patients were excluded because they did not have a confirmation of a haematological malignancy from their medical records. Ultimately, we included a total of ten patients after careful revision.

We determined the patients’ demographic and anamnestic (age, sex and comorbidities as represented by CCI) and microbiological, antimicrobial, and outcome data. The impact of infection was evaluated using clinical and microbiological data from medical records. Broad-spectrum antibiotics course were defined as molecules targeting both Gram-positive and Gram-negative bacterial groups such as broad-spectrum beta-lactams. Nosocomial pneumonia in our series is defined as pneumonia that develops within 48 h or more of hospital admission and which was not developing at the time of admission.

Isolates collected from various clinical specimens (rectal swabs, urine, blood, respiratory samples, etc.) were identified and tested for antimicrobial susceptibility (AST) using commercially available automated platforms (Vitek 2, bioMérieux, France and MicroScan WalkAway 96 Plus, Beckman Coulter, CA, USA). Minimum inhibitory concentration (MICs) results were confirmed, when necessary, by the Etest gradient diffusion method (bioMérieux). Cefiderocol AST was determined using disc diffusion method (Liofilchem, Italy) with a 30 µg disk incubated for 18–24 h at 35 ± 1 °C on Mueller–Hinton agar, as recommended by EUCAST. When necessary, zone diameter results were confirmed using lyophilised panels (Sensititre, Thermo Fisher Scientific, Waltham, MA, USA). TMP/SMX susceptibility data were interpreted according to *S. maltophilia*-specific breakpoints from the European Committee on Antimicrobial Susceptibility Testing (EUCAST). Levofloxacin and cefiderocol results were interpreted according to PK-PD (non-species-related) EUCAST breakpoints. 

### Statistical Analysis

A database for data collection was created using a Microsoft Excel table (2022 version), and we performed statistical data analysis with SPSS version 27 software. The descriptive analysis is reported as frequencies and percentages for categorical variables, and means and standard deviations for numeric variables.

## 3. Results

We reviewed medical records from 1 January 2021 to 1 June 2023. We found ten patients with *S. maltophilia* infection for inclusion in the present analysis. Table 1 shows the patients’ demographic and clinical characteristics. Patients from IRCCS Candiolo had CRBSI or bloodstream infection (BSI), whilst patients from City of Health and Sciences had only respiratory infections. 

The median age at admission was 67 years (interquartile range [IQR]: 49–76 years), and seven patients (70%) were males. The sample’s median Charlson Comorbidity Index (CCI) [12] was 6 (IQR: 4–8). Regarding the patients’ main haematological comorbidities, most of the adults were diagnosed with acute myeloid leukaemia (AML) (*n* = 3; 30%) and non-Hodgkin’s lymphoma (NHL) (*n* = 3; 30%). Three (30%) patients had undergone hematopoietic SCT before infection, all for AML, with a median time of 18 days (IQR: 14–22 days) between HSCT and infection. The most frequent comorbidities, other than haematological, were chronic lung diseases (*n* = 4; 40%), diabetes mellitus (*n* = 2; 20%) and cardiovascular diseases (*n* = 2; 20%). All the patients (N = 10; 100%) had undergone a recent broad-spectrum antibiotics course (30 days before infection) and had an indwelling central venous catheter before infection. Reflecting typical haematological complications after chemotherapy or SCT, we found prolonged neutropenia (>10 days) in eight patients (80%) and mucositis in four (40%). Only one patient in each group had a history of surgery or ICU admission with mechanical ventilation in the previous 90 days. In addition, eight patients (80%) reported a previous recent infection (<30 days) before *S. maltophilia* infection, as reported in Table 1. Screening rectal swabs were performed at admission and during hospitalisation before infection, and two patients tested positive for colonisation. Moreover, HSCT and AML patients were tested with oral swabs, and data were available for two patients.

The clinical presentation of infections constituted mainly nosocomial pneumonia (NP)—either with (2; 20%) or without (4; 40%) associated bloodstream infection (BSI)—and CRBSI (3; 30). Among patients with NP, two patients presented pulmonary haemorrhage (HP). NP in our series is defined as pneumonia that develops within 48 h or more of hospital admission and which was not developing at the time of admission. Moreover, NP complicated by HP was diagnosed by hemoptoe, using the radiological features of alveolar bleeding and the association in the literature between SM and this clinical presentation.

All three patients diagnosed with CRBSI presented a polymicrobial infection; the bacteria isolated with *S. maltophilia* are reported in Table 1. 

We collected microbiological susceptibilities of the isolated bacteria, as reported in Table 2.

Tetracycline and aminoglycoside susceptibilities were not available from either centre involved in the study. All tested strains for cefiderocol (7; 70%) were susceptible; all the samples were susceptible with increased exposure to TMP-SMX (100%), and no resistant strains were found.

Three (30%) patients were treated with TMP-SMX in monotherapy, and two (20%) with TMP-SMX in combination with cefiderocol. Three patients with strains susceptible to fluoroquinolones were treated with levofloxacin. Two patients underwent cefiderocol monotherapy. 

The median duration of antibacterial treatment was ten days (range: 7–22); the specific durations of each case are reported in Table 2. No adverse effects were reported. Clinical improvement was reported in 90% (*n* = 9) of the patients after initiating antibiotic treatment. Blood culture and lower respiratory tract surveillance samples were used to test for microbiological eradication of BSI and NP infections. Microbiological eradication was reported in 80% of the patients. Two patients, one with NP and another with NP and BSI, did not show microbiological eradication in the follow-up cultures.

Three patients reported infectious complications other than *S. maltophilia,* as reported in Table 2. Moreover, six patients reported complications other than infectious complications, especially vascular complications, such as acute bleeding or ischemic events (Table 2). The 30-day mortality rate was 30% (3 of 10), and the in-hospital mortality rate was 50% (5 of 10). In four of the five patients who ultimately died, vascular complications were identified as the principal cause of death.

## 4. Discussion

We report a case series of *S. maltophilia* infections among patients with high-risk haematological malignancies and aHSCT in two haematological centres. Moreover, amongst the full cohort of patients, four patients were treated with cefiderocol in monotherapy or in combination with other active antibiotics against intermediately susceptible strains of *S. maltophilia*. The 30-day mortality rate was 30%, and the in-hospital mortality rate reached 50%.

In our analysis, most of the patients were males (70%), in alignment with other retrospective studies in the haematological setting reviewed [3,4,6,9,14]. Tseng and colleagues [15] reported male gender as an independent risk factor for mortality in a group of patients affected by nosocomial pneumonias due to *S. maltophilia*. 

Moreover, the median age of 67 years in this cohort closely approximated the median ages reported by other authors, such as Kim et al. [16] and Karaba et al. [17], who reported median ages of 69 and 62 years, respectively. All of the BSIs and *S. maltophilia* infections in our report were diagnosed from blood culture harvested at least 72 h after admission. Chang et al. demonstrated that hospital-acquired *S. maltophilia* BSIs were characterised by a higher median age (65 vs. 59.2, *p* = 0.053) with respect to community-acquired infections [18].

We reviewed a population with high frequency of comorbidity; the median CCI was 6 (IQR, 4–8). Ebara et al. and Kim et al. in their works reported that between different groups, a higher CCI was an independent risk factor for mortality from *S. maltophilia* infections [5,19]. These data were recently confirmed by Appaneal et al. in a large cohort (N = 3891) of hospitalised patients treated for *S. maltophilia* infections [20]. In addition, median and IQR CCI levels in different haematological patients infected by *S. maltophilia* were lower than the data presented in our cohort [16,19,20].

AML (i.e., before or after HSCT) was the most commonly reported diagnosis (60%) in this study. In previous works in this field, AML was the first haematological malignancy reported, with a frequency between 32 and 68% [3,4,6,9] in patients with *S. maltophilia* infections. Patients with AML have an exceptionally high probability of experiencing unfavourable outcomes when *S. maltophilia* infections are diagnosed, with an overall mortality rate greater than 20% in patients with primary bacteremia and greater than 60% in patients with pneumonia [20].

Regarding non-haematological comorbidities, we uncovered a high prevalence of chronic lung diseases. *S. maltophilia* frequently colonised the respiratory tracts of patients with known chronic lung diseases, particularly cystic fibrosis and chronic obstructive pulmonary diseases [21]. More recently, in a systematic review, Wang et al. [22] defined risk factors for lower respiratory tract infections due to *S. maltophilia*, such as severe illness, high APACHE-II scores, invasive procedures, broad-spectrum antibiotics, impaired immune function and long-term hospitalization [22]. These risk factors are also not uncommonly reported in the haematological population that is prone to invasive procedures, long-term hospitalisation, and broad-spectrum antibiotic courses.

Moreover, cardiovascular diseases and diabetes mellitus were common comorbidities in our cohort, in alignment with research previously reported by different groups [4,6,9].

All the patients in our case series reported a recent history of a broad-spectrum antimicrobial regimen in the 30 days prior to their *S. maltophilia* infection. In a recent systematic review and meta-analysis, Wang et al. found that beta-lactamase inhibitors (OR = 9.98, 95% CI: 1.51~65.96) and carbapenems (OR = 2.82, 95% CI: 1.49~5.31) were independent risk factors in relation to subsequent *S. maltophilia* infections in the ICU [22]. In a case–control study of oncological patients, Apisarnthanarak et al. found, amongst cases concerning controls, a higher median number (i.e., 9 vs. 5) of antibiotics used (*p* < 0.001) in the days before an invasive *S. maltophilia* infection [23]. Moreover, the authors [23] reported among significant risk factors for *S. maltophilia* bacteremia the presence of mucositis (7 [53.8%] of 13 vs. 8 [20.5%] of 39; *p* =0.034), which was also reported frequently (40%) in our cohort. La Barca et al. [24] reported findings regarding HSCT patients in a case–control study (cases were patients for whom hospitalisation was complicated by a *S. maltophilia* infection). The authors [24] found that the presence of severe neutropenia (*p* = 0.028) and a longer duration of severe neutropenia (*p* = 0.05) or severe mucositis (*p* = 0.028) increased the risk of *S. maltophilia* infections. Neutropenia, mucositis and long-term use of antimicrobial therapies or broad-spectrum antibiotic courses were also reported in a notable review by Al-anazi et al. regarding risk factors for pneumonia and BSI due to *S. maltophilia* infections within the HSCT population [25].

Most patients reported a previous bacterial, fungal or viral infection. Amongst viral infections, SARS-CoV-2 and influenza were reported as commonly viral co-infections in patients with *S. maltophilia* subsequent infectious complications [25]. In our series, *S. maltophilia* complicated the hospitalisation of these patients after the end of treatment and the clinical course of viral illnesses. Nevertheless, infectious complications that occurred before *S. maltophilia* strongly contributed to antimicrobial use, length of hospitalisation and arguably the morbidity and mortality rates that manifested in this cohort.

We found that 20 to 33.3% of patients carried *S. maltophilia* on their rectal or oral swabs before contracting invasive infections. Gut colonisation and particularly oral colonisation by *S. maltophilia* were significantly associated with subsequent invasive infections, particularly in haematological patients treated via prolonged antibiotic use [26,27]. Therefore, to identify patients who may benefit from early, effective therapy for *S. maltophilia*, a risk score for the acquisition of *S. maltophilia* BSI in the haematological malignancy population was recently created by Karaba et al. [17]; AML, absolute neutrophil count category, oncologist-diagnosed mucositis, the presence of a central venous catheter, and at least three days of carbapenem treatment within the preceding three months were all components of this score.

Most of the patients (6/10) in our case series developed pneumonia with or without associated BSI, and secondly, CRBSI (3/10). Pneumonia was rarely reported as the first clinical presentation of *S. maltophilia* diseases, and the most frequent primary focus was CRBSI in most cases [20,28,29,30]. Moreover, we found that, in two patients, pneumonias were the source of *S. maltophilia* bacteremia. Lai et al. [31] found that while *S. maltophilia* bacteremia originating from the respiratory tract as a starting point was associated with higher mortality, CVC-related bacteremia was inversely associated with mortality. In addition, Boktour et al. found that secondary bacteremia had a more severe prognosis than primary catheter-related bacteremia [32]. The lower mortality reported for CRBSI could be related to the possibility of a quick removal of the infection source by removing the device at the time of microbiological diagnosis. 

In patients diagnosed with CRBSI, we found a polymicrobial infection in all three cases, as reported in Table 1. It is not uncommon for *S. maltophilia* to be found in polymicrobial infections; the rate at which this pathogen is isolated as part of a mixed infection varies from 33% to 70% [33]. Some of the bacterial species reported in our series, particularly coagulase-negative *Staphylococcus*, *Escherichia coli* and *Pseudomonas aeruginosa*, have commonly been detected in the literature regarding polymicrobyal infections, along with *S. maltophilia* [33]. Since other more virulent organisms may play a larger role in these infections, determining *S. maltophilia*’s pathogenic involvement is challenging. Patients with monomicrobial *S. maltophilia* bacteraemia had higher death rates than those with polymicrobial bloodstream infections, according to a recent retrospective study that analysed 10 years’ worth of data regarding *S. maltophilia* bacteraemia amongst hospitalised adults at the Mayo Clinic Hospital in the United States [34]. Nonetheless, numerous investigations have concurred regarding the substantial reduction in mortality risk when *S. maltophilia*-active medication was commenced empirically, regardless of the type of infection (mono- or polymicrobial) [33].

Among the *S. maltophilia* strains collected, we found susceptibilities to cefiderocol in all the samples tested. Nakamura and colleagues [35] investigated the in vitro and in vivo activity of cefiderocol in either resistant or susceptible strains of *S. maltophilia* to TMP/SMX, showing a significant effect in both presented groups. Moreover, Biagi et al. reported data regarding susceptibilities of cefiderocol in TMP/SMX and/or fluoroquinolones in 37 strains of *S. maltophilia*; the susceptibility rate of cefiderocol was 100% [11]. This new siderophore cephalosporin could be an interesting weapon against *S. maltophilia* strains that are resistant to TMP/SMX and/or fluoroquinolones, or in patients with a high risk of adverse events or the worsening of pre-existent morbidities due to first-line treatment (i.e., kidney failure or bone marrow suppression with TMP/SMX or vascular disease, seizures, and QT elongation with fluoroquinolones).

Interestingly, between the two centres involved in the study, we found differences among fluoroquinolone susceptibilities in *S. maltophilia* strains. In the centre (Molinette hospital), we found a higher rate of strains susceptible to fluoroquinolones compared to the second centre (IRCCS Candiolo), but without statistical significance. We hypothesize that the epidemiology of the two centres diverges in part due to the differences in prophylaxis protocols. In the first centre, antibacterial prophylaxis with fluoroquinolones was not routinely used over the last three years, as reported in recent studies [36,37], while in the second, prophylaxis was still maintained in patients with AML or who had undergone aHSCT.

At the time of writing, the scientific community is lacking randomized controlled trials comparing TMP/SMX, the drug of choice for *S. maltophilia*, with other available molecules [33]. In addition, despite the risk profile of *S. maltophilia* regimens, no drug-related adverse events were reported in our series.

Despite a high rate of clinical improvement (90%) after antimicrobial therapy starting, we faced high 30-day mortality (30%) and in-hospital mortality (50%) rates. The reported estimates of mortality after *S. maltophilia* infections are primarily drawn from retrospective studies conducted at a single centre. These estimates range from 18% to 69% for all-cause death at various timepoints after infection, and they range from 24% to 58% for attributable mortality [38,39,40]. Unfortunately, we reported two cases of HP that contribute to these mortality rates. This clinical presentation is the most lethal type of *S. maltophilia* infection, with a case fatality rate close to 100% [33].

The difference between crude mortality and mortality attributable to *S. maltophilia* remains unclear in many aspects. In our series, vascular complications (i.e., bleedings and ischemic diseases) were directly involved in the outcome of our small group of patients, in association with high comorbidities at baseline.

There were some limitations to this case series. First, it is a report regarding two centres with a small sample size, which may not be generalizable. Second, it is a case series, and due to the nature of the manuscript, statistical tests yielding *p* values or confidence intervals were not generally used. Third, the cause of mortality was not consistently documented, and had to be inferred in some cases. Moreover, medical records were revised independently by two authors, and the decision to treat *S. maltophilia* infections was made by three authors, based on criteria included in Material and Methods section. The distinction between colonization and infection with *S. maltophilia* was evaluated through the revision of clinical history and radiological, laboratory, and microbiological features agreed to strongly suggest *S. maltophilia*.

## 5. Conclusions

The mortality rate of S. maltophilia infection is reported in the literature to be from 18% to 69% for all-cause death at various timepoints after infection, and ranges from 24% to 58% for attributable mortality. Moreover, the baseline characteristics and comorbidities of haematological population mean they carry a high risk of complications and related mortality, which is probably not directly linked to the infectious complications themselves. In this cohort, we describe a small sample of haematological patients, including HSCT patients affected by *S. maltophilia* infections, notably NP. 

Interestingly, we reported our first four cases treated with cefiderocol without adverse effects. In conclusion, *S. maltophilia* infection in the haematological population results in a high rate of mortality.

## Figures and Tables

**Table 1:** Demographics and clinical characteristics of the population.

Characteristics	N (%)
Sex (Female)	3 (30)
Age (Median, Range)	67 (49–76)
CCI (Median, Range)	6 (4–8)
Comorbidities and Risk Factors	
Haematological disease	10 (100)
AML	3 (30)
NHL	3 (30)
aHSCT for AML	3 (30)
HL	1 (10)
Cephalosporins, beta-lactams, beta-lactams with beta-lactam inhibitors or carbapenems antibiotic therapy (<30 days)	10 (100)
Fluoroquinolones prophylaxis	4 (40)
Indwelling CVC before SM	10 (100)
Prolonged (>10 days) Neutropenia before SM	8 (80)
Mucositis	4 (10)
Chronic obstructive pulmonary diseases	4 (10)
Cardiovascular diseases	2 (20)
Diabetes mellitus	2 (20)
Obesity	1 (10)
Surgery <90 days	1 (10)
ICU <90 Days with MV	1 (10)
Days from HSCT before SM (days)	18 (14–22)
LOS before SM infection (days)	32 (22–44)
Other infections in the 30 days beforeSM	
Gram-negative BSI	
E.coli ESBL	1 (10)
P. aeruginosa Amp-C-producing	1 (10)
Gram-positive BSI	
MRSE	1 (10)
C. difficile colitis	1 (10)
Fungal Respiratory Tract Infections	
PJP	1 (10)
Viral Respiratory Tract Infections	
Metapneumovirus bronchiolitis	1 (10)
COVID-19	1 (10)
Influenza pneumonia	1 (10)
Rectal or Oral Swabs at Admission	
SM Rectal Carriage	2 (20)
SM Oral Carriage	2/6 (33.33%)
Type of SM Infection	
NP	2 (20)
HP	2 (20)
NP + BSI	2 (20)
Polymicrobial CRBSI	3 (30)
E. coli NDM	1 (10)
MRSE, Achromobacter, P. aeruginosa	1 (10)
MRSE, P. aeruginosa	1 (10)
Monomicrobial BSI	1 (10)
Septic shock (SEPSIS-3 criteria) at presentation	4 (10)
Source Control	
Yes	3 (30)
No or Not Feasible	7 (70)

Abbreviations: CCI: Charlson comorbidity index; N: number; AML: Acute myeloid leukemia; NHL: Non-Hodgkin’s lymphoma; HL: Hodgkin’s lymphoma; NA: not available; aHSCT: allogenic hematopoietic stem cell transplant; CVC: central venous catheter; SM: *Stenotrophomonas maltophilia*; ICU: intensive care unit; MV: mechanical ventilation; LOS: length of hospital stay; ESBL: extended-spectrum beta-lactamases; MRSE: methicillin-resistant *Staphylococcus epidermidis*; NP: nosocomial pneumonia; BSI: bloodstream infection; CRBSI: catheter-related bloodstream infection; HP: haemorragic pneumonia; NDM: New-Delhi Metallo-beta-lactamases.

**Table 2:** Microbiological susceptibilities, clinical presentations, outcomes and complications.

Patient	Cefiderocol	TMP/SMX	Levofloxacin	Treatment Option	Duration of Treatment	Adverse Effect	Type of Infection	Specimen	Septic Shock	Clinical Improvement	Microbiological Eradication	30-Day Mortality	In-Hospital Mortality	Other Subsequent Infections	Other Complications
1	S	I	R	Cefiderocol	22	No	NP + BSI	BAL, blood	Yes	Yes	Yes	0	0	BK virus cystitis	Skin GVHD
2	S	I	R	Cefiderocol + TMP/SMX	14	No	HP	BAL	Yes	Yes	No	0	1	No	Intra-alveolar bleeding (Haemorrhagic pneumonia)
3	S	I	R	Cefiderocol	18	No	HP	BAL	No	Yes	Yes	0	1	No	Stroke
4	S	I	R	Cefiderocol + TMP/SMX	14	No	CRBSI	Blood	Yes	Yes	Yes	0	0	Pulmonary toxocariasis	No
5	NA	I	S	Levofloxacin	10	No	CRBSI	Blood	No	Yes	Yes	0	0	COVID-19	No
6	NA	I	R	TMP/SMX	10	No	BSI	Blood	No	Yes	Yes	1	1	No	Gastro-Intestinal bleeding
7	S	I	S	Levofloxacin	10	No	CRBSI	Blood	No	Yes	Yes	0	0	No	No
8	NA	I	S	TMP/SMX	10	No	NP	BAL	No	Yes	Yes	1	1	No	Intra-alveolar bleeding (Haemorrhagic pneumonia)
9	S	I	S	Levofloxacin	7	No	NP	BAL	No	Yes	Yes	0	0	No	No
10	S	I	NA	TMP/SMX	7	No	NP + BSI	BAL, blood	Yes	No	No	1	1	No	No

Abbreviations: NA: not available; R: resistant; I: susceptible, increased exposure; S: susceptible; TMP/SMX: Trimethoprim/sulfametoxazole; NP: nosocomial pneumonia; BSI: bloodstream infection; CRBSI: catheter-related bloodstream infection; HP: haemorragic pneumonia; GVHD: graft versus host disease. BAL: broncho-alveolar lavage.

## Data Availability

Data are available from the authors upon request.
